# Genus-Wide Characterization of Nuclear Mitochondrial DNAs in Bumblebee (Hymenoptera: Apidae) Genomes

**DOI:** 10.3390/insects12110963

**Published:** 2021-10-23

**Authors:** Lele Ding, Huiling Sang, Cheng Sun

**Affiliations:** Institute of Apiculture Research, Chinese Academy of Agricultural Sciences, Beijing 100093, China; dingleledinglele@163.com (L.D.); sanghuiling2020@163.com (H.S.)

**Keywords:** bumblebee, mitogenome, NUMT, genome innovation

## Abstract

**Simple Summary:**

The DNA of mitochondria can be transferred into the nucleus and form nuclear mitochondrial DNAs (NUMTs). In this study, we identified and characterized NUMTs in genus-wide bumblebee species. The number of NUMTs in bumblebee is much lower than those in its closely related taxon, honeybee. The insertion sites of NUMTs in bumblebee are not random, with AT-rich regions harboring more NUMTs. In addition, NUMTs derived from the mitochondrial COX1 gene are most abundant in the nuclear genome. While the majority of NUMTs seem unfunctional in the bumblebee, some NUMTs show functional clues, which could fuse with their flanking sequences to form novel proteins. Our results shed light on the molecular features of NUMTs and uncover their contribution to genome innovation in the bumblebee.

**Abstract:**

In eukaryotes, DNA of mitochondria is transferred into the nucleus and forms nuclear mitochondrial DNAs (NUMTs). Taking advantage of the abundant genomic resources for bumblebees, in this study, we de novo generated mitochondrial genomes (mitogenomes) for 11 bumblebee species. Then, we identified and characterized NUMTs in genus-wide bumblebee species. The number of identified NUMTs varies across those species, with numbers ranging from 32 to 72, and nuclear genome size is not positively related to NUMT number. The insertion sites of NUMTs in the nuclear genome are not random, with AT-rich regions harboring more NUMTs. In addition, our results suggest that NUMTs derived from the mitochondrial COX1 gene are most abundant in the bumblebee nuclear genome. Although the majority of NUMTs are found within intergenic regions, some NUMTs do reside within genic regions. Transcripts that contain both the NUMT sequence and its flanking non-NUMT sequences could be found in the bumblebee transcriptome, suggesting a potential domestication of NUMTs in the bumblebee. Taken together, our results shed light on the molecular features of NUMTs in the bumblebee and uncover their contribution to genome innovation.

## 1. Introduction

In eukaryotes, the DNA of mitochondria can be transferred to the nuclear genome and form so-called nuclear mitochondrial DNAs (NUMTs) [1,2,3,4,5,6]. NUMTs are inserted into the nuclear genome during the repair of double-strand breaks (DSBs) by the non-homologous end-joining repair (NHEJ) pathway [4,7]. The number of NUMTs varies across species, and broadly, three reasons could underlie such variation: (1) the frequency of DSBs is different across species; (2) the frequency of DSBs repaired by NHEJ vary between species; and (3) the rate of NUMT loss is different across species [3,5,8]. After integration into the nuclear genome, NUMTs are believed to be mainly nonfunctional sequences, which could also disrupt nuclear functional genes (occasionally causing human genetic disease) or contribute beneficially to gene evolution [9,10,11]. The presence of NUMTs could be problematic when using mitochondrial DNA (mtDNA) as molecular markers in evolutionary studies; if mtDNA is amplified by PCRs using common methodology, NUMTs could be co-amplified, which will mislead downstream phylogenetic analysis [12].

Bumblebees (Hymenoptera: Apidae) are a group of insects that comprise the genus *Bombus*, with ~250 extant species classified into 15 subgenera [13,14,15]. They are important pollinators for both wild flowers and crops [16,17]. Identification and characterization of NUMTs in bumblebees will not only uncover their molecular features (i.e., abundance, diversity, and evolution) and thus provide a general idea on to what extent NUMTs will affect phylogenetic analysis when using mtDNA as molecular markers in the bumblebee but will also shed light on the contribution of NUMTs to bumblebee gene and genome evolution. However, although the presence of NUMTs in the bumblebee has been implicated in a previous study [18], no detailed, genome-wide analysis has yet been conducted. Taking advantage of the genus-wide bumblebee genomic resources [19,20], we performed a systematic examination of NUMTs in bumblebees to infer their abundance, origin, and evolution. Our study sheds light on the molecular features of NUMTs in bumblebee, as well as their contribution to genome innovation.

## 2. Materials and Methods

For bumblebee species that already have a reported mitogenome, their mitogenome sequences were downloaded from GenBank. For species that do not yet have a mitogenome, their Illumina shotgun reads for whole-genome sequencing were downloaded from NCBI Sequence Read Archive (SRA) (https://www.ncbi.nlm.nih.gov/sra (accessed on 28 August 2020)). The accession numbers for mitogenome sequences and Illumina shotgun reads are summarized in Table 1. The genomic sequences and genome annotation of *B. terrestris* were downloaded from GenBank (assembly accession: GCF_000214255.1). The genome assemblies and annotations for all the other bumblebees were downloaded from the National Genomics Data Center (https://ngdc.cncb.ac.cn (accessed on 28 August 2020)), under BioProject number PRJCA002292.

To generate mitogenome sequences, the downloaded Illumina shotgun reads of each species were assembled by NOVOPlasty v2.6.3 [21], MitoZ v2.4-alpha [22] and GetOrganelle v1.7.1 [23] software. When running NOVOPlasty, we used the *B. terrestris* COX1 gene as seed; when running MitoZ, parameters “--genetic_code 5 --clade Arthropoda --run_mode 2 --filter_taxa_method 1 --requiring_taxa ‘Arthropoda’” were applied; when running GetOrganelle, parameters “-R 10 -k 21,45,65,85,105 -F animal_mt” were used; all other parameters were set as default. The resulting mitogenomes were analyzed by using the MITOS web server [24]. Manual merging assisted by NCBI-BLAST2 was performed when one software generated more than one contigs and none of them contain all of the 13 mitochondrial protein-coding genes. The most complete mitogenomes (containing the most protein-coding and RNA genes—if the gene numbers were the same, then the longest sequence) obtained from the three assemblers were kept for each species for downstream analysis.

Bumblebee genomes are rich in AT [20]. To minimize false positives, NUMTs were identified by using mitogenome sequences as queries to perform BLASTn (-task blastn) against their respective nuclear genome sequences with an e-value cutoff of 6 × 10^−14^, a strict threshold that has previously been applied in NUMT analysis in the honeybee genome [25]. Identified NUMTs that are located at the ends of each scaffold were removed from downstream analysis.

The coordinates of NUMTs were used to calculate their lengths, which were also compared with the coordinates of protein-coding genes of the same genome to infer the relative positions of NUMTs to its nearest genes. Then 100 bp of upstream and downstream sequences of NUMTs in each genome were extracted, which were then analyzed by Genetyx7 software (GENETYX CORPORATION) to determine their AT content.

The correlation between NUMT number and mitogenome length, or nuclear genome length, was calculated by “cor.test ()” function in R (http://www.R-project.org/ (accessed on 28 August 2020)).

For bumblebee species that have available RNA-seq data (Table 1) [19], shotgun reads were assembled using Trinity [26] with default parameters. For *B. terrestris*, whose genome has been intensively annotated, we used its curated transcripts directly (assembly accession: GCF_000214255.1). To identity chimeric transcripts that contain both NUMT sequence and flanking non-NUMT sequences, which suggests the potential domestication of NUMT (serving a novel function in the nuclear genome), the sequences of NUMT, along with its 60 bp of flanking sequences, were extracted and used as queries for BLASTn against the assembled transcriptome sequence of each species, with an e-value cutoff of 1 × 10^−10^ and a sequence identity cutoff of 0.98. Transcripts that contain an NUMT sequence and at least 50 bp of its flanking non-NUMT sequences were eligible chimeras.

## 3. Results

### 3.1. Genus-Wide Mitogenome Resources for Bumblebees

In this study, we de novo generated mitogenomes for 11 bumblebee species, whose mitogenome sizes range from 15,029 to 29,809 bp, with an average length of 17,839 bp (Table 2). These mitogenomes are complete or almost complete: all of them have 13 protein-coding genes, and most of them have two rRNA genes and 22 tRNA genes (Table 2). Notably, *B. sibiricus*, which has the largest nuclear genome size across genus-wide species, also has the largest mitogenome size (Table 2). Coupled with previously reported bumblebee mitogenomes [27,28,29,30,31], we obtained mitogenomes for species spanning all of the 15 *Bombus* subgenera.

### 3.2. Molecular Features of NUMTs in Bumblebee Nuclear Genomes

**Figure 1:**
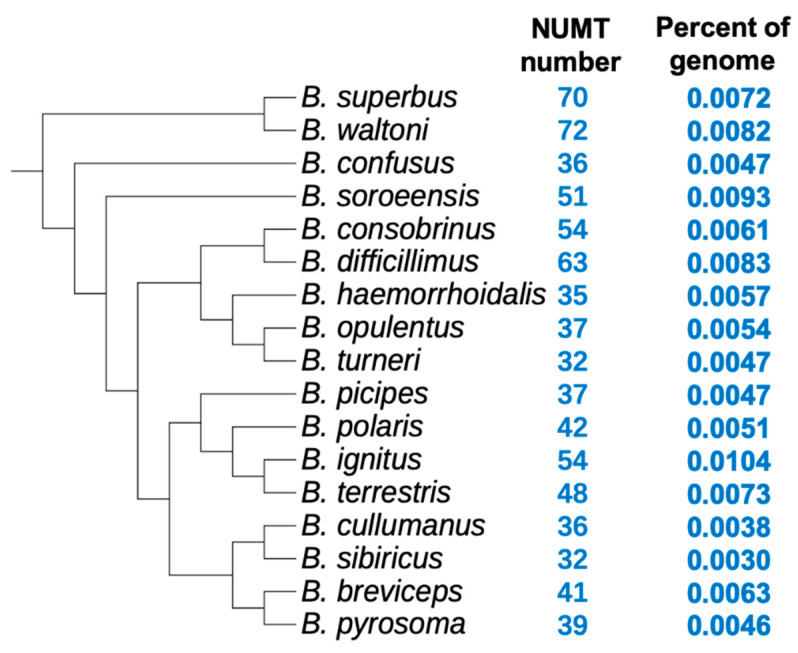
NUMTs identified in each of the 17 bumblebee species. The phylogeny was deduced from a previous study [19].

A total of 17 species were searched for NUMTs, and the results are summarized in Figure 1. From the results, we could see that NUMTs vary in number across species, ranging from 32 to 72. The NUMT counts in the bumblebee are much lower than those in its closely related taxon honeybee, where more than 1000 NUMTs could be identified [25,32]. Regarding sequence length, NUMTs only comprised a tiny portion, ranging from 0.0030% to 0.0104% for each nuclear genome (Figure 1).

**Figure 2:**
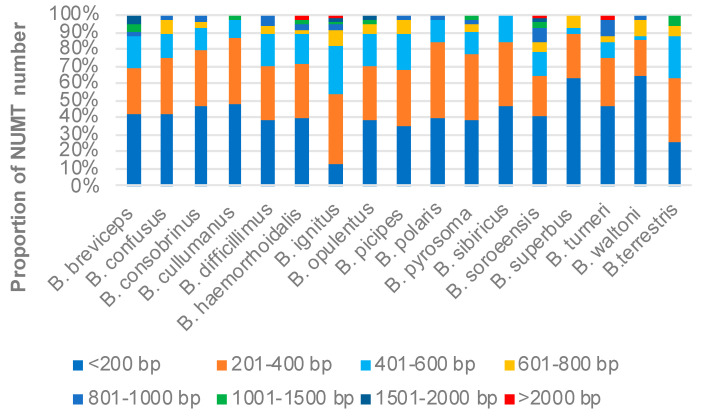
Length distribution of NUMTs in bumblebee.

The length distribution of the NUMTs is summarized in Figure 2, from which we can observe that, while the majority of bumblebee NUMTs are less than 400 bp in length, NUMTs longer than 2000 bp could also be found in some species.

**Figure 3:**
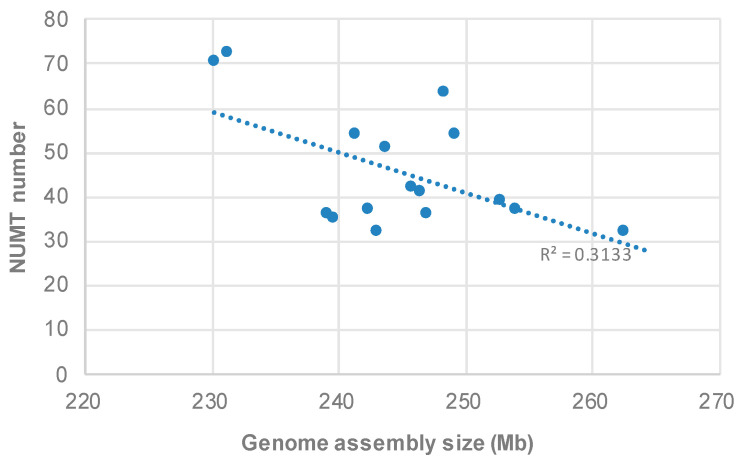
Relationship between NUMT number and nuclear genome size. Genome assembly sizes are from previous reports [19].

To understand factors that contribute to the variable number of NUMTs in different bumblebee species, firstly, we investigated the relationship between NUMT numbers and the length of mitogenomes. The results suggested that the variation of NUMT number is not correlated with mitogenome length (R^2^ = 0.0498; *p*-value = 0.3891), ruling out the possibility that the completeness of mitogenome may underlie such variation. Then, we correlated NUMT number with nuclear genome size, and, unexpectedly, we identified a negative correlation between them (R^2^ = 0.3133; *p*-value = 0.02188; Figure 3). When we related the nuclear genome size with the total sequence length of NUMTs, no statistically significant correlation was identified (*p*-value > 0.05).

**Figure 4:**
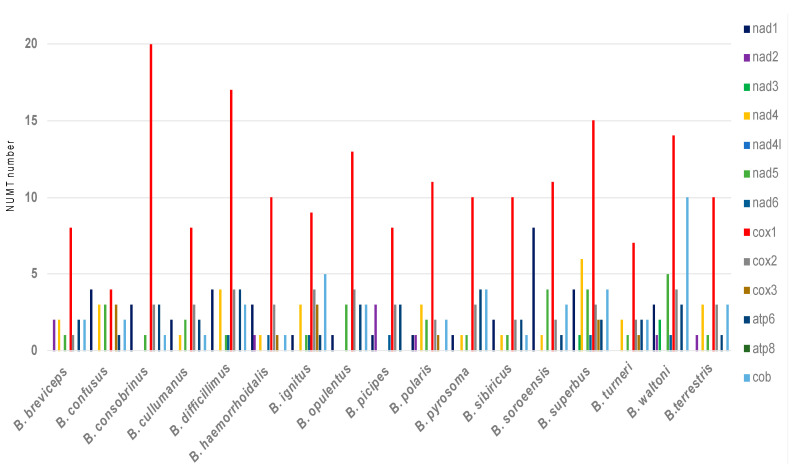
Number of NUMTs derived from each of the 13 mitochondrial protein-coding genes.

To understand the origin of NUMTs, we tracked NUMTs back to their respective mitogenomes. The numbers of NUMTs that were derived from each of the 13 mitochondrial coding genes were summarized for each species, and the results indicated that, in all of the 17 species, COX1-derived NUMTs are most frequently present in the nuclear genome (Figure 4). Our finding is similar to those in reports regarding the honey bee [25,32] and yeast [33].

**Figure 5:**
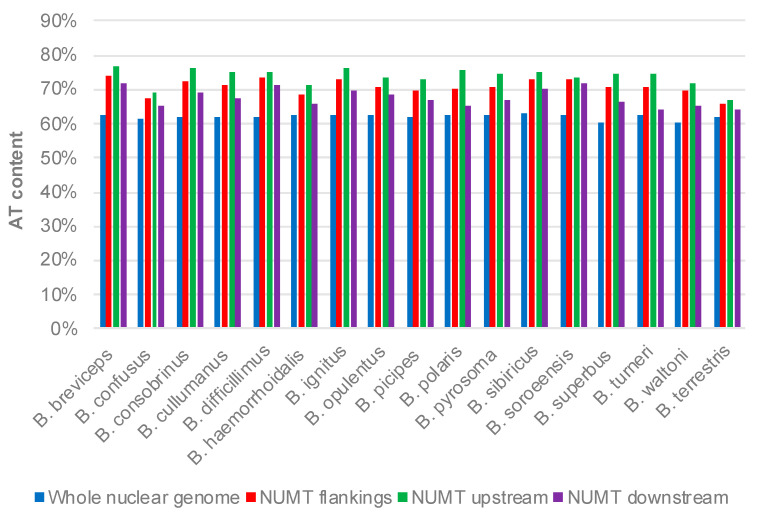
AT contents of the NUMT flanking sequences and whole nuclear genome.

To understand the sequence preference of NUMT insertion, we extracted 100 bp of flanking sequences of NUMTs and investigated their sequence composition. Our results suggested that, in all of the 17 species, the AT contents of NUMT flanking sequences as a whole, upstream flanking sequences, and downstream flanking sequences are all higher than those of the whole nuclear genome (Figure 5), indicating a preference of the AT-rich sequence for NUMT insertion in the bumblebee nuclear genome. The preference of NUMTs for AT-rich regions has also been reported in fig wasp genomes [34]. Notably, the AT content of the NUMT upstream flanking sequence is consistently higher than that of the downstream flanking sequence in all of the 17 species (Figure 5).

### 3.3. Contribution of NUMTs to Bumblebee Genome Innovation

**Figure 6:**
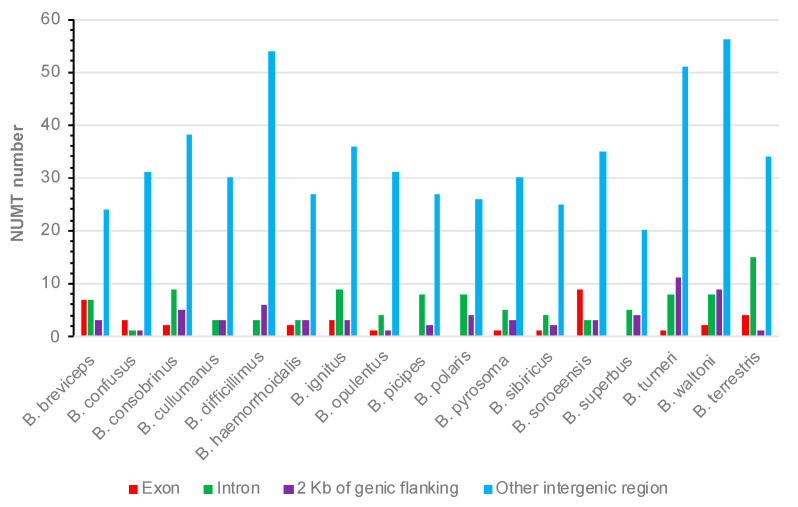
Distribution of NUMTs in relation to their nearest nuclear protein-coding genes.

To understand if NUMTs could contribute to nuclear genome innovation in the bumblebee, the distribution of NUMTs in relation to nuclear protein-coding genes was analyzed. The results indicated that, in all of the 17 species, the majority of NUMTs were located within the intergenic regions (Figure 6). However, NUMTs, with numbers varying from 5 to 20 across species, were found within genic regions (exons and introns) or their 2 Kb of flanking (Figure 6). Considering their proximity to protein-coding genes, such NUMTs have the possibility of being domesticated in the nuclear genome.

**Figure 7:**
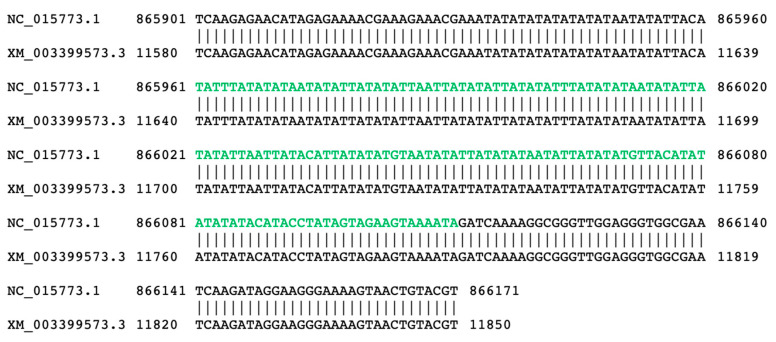
Domestication of one NUMT in bumblebee. Alignment between one genomic region of *B. terrestris* that harbors NUMT and one transcript that could support the transcription of NUMT. Characters in green indicate NUMT sequence. NC_015773.1 and XM_003399573.3 indicate the NCBI accession numbers for genomic sequence and transcript, respectively, with numbers flanking pairwise alignments indicating coordinates in each respective sequence. Based on the annotation report from NCBI, transcript XM_003399573.3 could be 100% covered by RNA-seq alignments.

To identify functional clues for those NUMTs, we identified NUMTs that fused with their flanking non-NUMT sequences and formed chimeras, for which we could find transcript evidence. Transcripts that contain the NUMT sequence and at least 50 bp of its flanking non-NUMT sequences could be found in all of the 13 species, for which we have transcriptome data, with numbers ranging from 5 to 18 (Table 3). Here, we show one such example: in the *B. terrestris* gene mucin-5AC, one NUMT fused with its flanking sequences and formed a new functional exon (Figure 7). Therefore, our results suggest that some NUMTs might have been domesticated in the nuclear genome and contribute to nuclear genome innovation in the bumblebee.

## 4. Discussion

In this study, using publicly available genomic resources and three popular software packages, we de novo generated mitogenome sequences for 11 bumblebee species (Table 2). To this end, we obtained mitogenomes for species spanning all of the 15 *Bombus* subgenera, which will facilitate studies on the evolution of mitogenomes, as well as the interaction between mitogenome and nuclear genome, in the bumblebee.

Some molecular features of NUMTs are conserved across genus-wide bumblebee species. For example, NUMTs derived from the mitochondrial COX1 gene are most abundant in all of the 17 analyzed bumblebee nuclear genomes (Figure 4). In the honeybee, the closely related taxa of the bumblebee, similar findings have been reported, which might be explained by the faster migration rate of this mtDNA region to the nucleus than that of the other mtDNA regions [25]. Our results also indicated that NUMTs are preferably present in AT-rich regions of the nuclear genome in all of the 17 focal species, and the AT content of NUMT upstream sequences is consistently higher than that of downstream sequences (Figure 5). The preference of AT-rich regions for bumblebee NUMTs is similar to the finding reported for humans, but no such preference has been identified in the honeybee [10,32].

The number of NUMTs considerably varies across the 17 analyzed species (Figure 1). Many factors have been proposed to affect the abundance of NUMTs, and when different sample sizes are used, different conclusions are obtained [3,5,8]. Comparative analysis of NUMTs in multiple closely related species, which could narrow down the genetic background difference between species, may provide new insights into factors underlying the variation of NUMT abundance. As NUMTs are integrated passively into the nuclear genome during the repair process of double-strand breaks (DSBs), we could expect that the more DSBs in one nuclear genome, the more NUMT insertions [2,3,4,7]. In yeast, the number of DSBs proportionally increased as the amount of nuclear DNA increased [35]; thus, if this trend is a general rule, then larger genomes will have more DSBs and, therefore, will have more NUMTs [3]. However, when we correlated nuclear genome sizes with NUMT counts across bumblebee species, we did not find such a positive correlation (instead, a negative correlation was observed) (Figure 3). In addition, when relating the nuclear genome size with the total sequence length of NUMTs, no statistically significant correlation was identified (*p*-value > 0.05). Therefore, our results suggested that larger nuclear genomes, which should have more DSBs, do not necessarily harbor more NUMTs in the bumblebee. Future studies could investigate if the rate of NUMT loss or the frequency of DSBs repaired by NHEJ is different across bumblebee species, as these two factors could also contribute to the variable number of NUMTs across species [3,5,8].

NUMTs are believed to be mainly nonfunctional sequences and could also cause disease or beneficially contribute to gene evolution [9,10,11]. In this study, we found that, while the majority of NUMTs were located within intergenic regions, there were NUMTs that reside within genic regions (Figure 6). In addition, we could identify transcripts that contain both NUMTs and their immediate flanking sequences, with numbers ranging from 5 to 18 across species, indicating that these NUMTs might have been domesticated in the nuclear genome to form functional exon sequences (Table 3; Figure 7). Here we used 50 bp of flanking as a threshold to observe as many potential domestication events as possible, but such criteria may contain false positives, as chimeric RNA-seq reads could be generated during Illumina library preparation/sequencing. Taken together, our results in this study suggest that—other than ‘junk’ sequences—NUMTs could contribute to nuclear genome innovation in the bumblebee, which invites further experimental validation.

## 5. Conclusions

In this study, we performed comparative analysis of NUMTs across genus-wide bumblebee genomes. Our results indicated that NUMT number varies across bumblebee species, but nuclear genome size is not positively related to NUMT number. NUMTs have a preference of AT-rich regions in bumblebee nuclear genome, and NUMTs derived from the mitochondrial COX1 gene are most abundant. Although the majority of NUMTs seem unfunctional, we identified evidence that some NUMTs should have been domesticated in the nuclear genome to form functional exon sequences. Taken together, our results shed light on the molecular features of NUMTs in the bumblebee nuclear genome and uncover their contribution to genome innovation.

## Figures and Tables

**Table 1:** Sequences used in this study and their accession numbers.

Species	Genomic Shotgun Reads	Published Mitogenomes	RNA-Seq
*B. breviceps*		MF478986	SRR12527963
*B. confusus*	SRR12527987		
*B. consobrinus*		MF995069	SRR12527965
*B. cullumanus*	SRR12528003		
*B. difficillimus*	SRR12527947		SRR12527962
*B. haemorrhoidalis*	SRR12527992		SRR12527929
*B. ignitus*		DQ870926	SRR12527935
*B. opulentus*	SRR12527970		SRR12527964
*B. picipes*	SRR12528031		SRR12527936
*B. polaris*	SRR12527998		
*B. pyrosoma*		MH998260	SRR12527934
*B. sibiricus*	SRR12527981		SRR12527966
*B. soroeensis*	SRR12528009		
*B. superbus*	SRR12528014		SRR12527932
*B. turneri*	SRR12528020		SRR12527933
*B. waltoni*		MK252702	SRR12527961
*B. terrestris*		KT368150	

**Table 2:** Statistics for the 11 newly generated mitogenome sequences.

Species	Total Length (bp)	Protein-Coding Gene	rRNA Gene	tRNA Gene
*B. confusus*	19,550	13	2	19
*B. cullumanus*	16,418	13	2	20
*B. difficillimus*	19,401	13	2	22
*B. haemorrhoidalis*	15,358	13	2	22
*B. opulentus*	17,293	13	1	20
*B. picipes*	18,381	13	2	21
*B. polaris*	16,785	13	2	22
*B. sibiricus*	29,809	13	2	21
*B. soroeensis*	17,138	13	2	22
*B. superbus*	15,792	13	2	22
*B. turneri*	15,029	13	2	19

**Table 3:** Summary of the fusion of NUMTs with their flanking sequences.

Species	Number of NUMTs Fused with Their Flanking Sequences
*B. breviceps*	18
*B. consobrinus*	8
*B. difficillimus*	9
*B. haemorrhoidalis*	9
*B. ignitus*	7
*B. opulentus*	7
*B. picipes*	8
*B. pyrosoma*	5
*B. sibiricus*	7
*B. superbus*	9
*B. turneri*	6
*B. waltoni*	12
*B. terrestris*	6

## Data Availability

Mitogenomes generated in the study have been deposited in GenBank at the National Center for Biotechnology Information (NCBI), with accession numbers MZ352138-MZ352148.
